# Dual-targeting anti-angiogenic cyclic peptides as potential drug leads for cancer therapy

**DOI:** 10.1038/srep35347

**Published:** 2016-10-13

**Authors:** Lai Yue Chan, David J. Craik, Norelle L. Daly

**Affiliations:** 1 The University of Queensland, Institute for Molecular Bioscience, 4072 Brisbane, Australia; 2 Centre for Biodiscovery and Molecular Development of Therapeutics, Australian Institute of Tropical Health and Medicine, James Cook University, 4870 Cairns, Australia

## Abstract

Peptide analogues derived from bioactive hormones such as somatostatin or certain growth factors have great potential as angiogenesis inhibitors for cancer applications. In an attempt to combat emerging drug resistance many FDA-approved anti-angiogenesis therapies are co-administered with cytotoxic drugs as a combination therapy to target multiple signaling pathways of cancers. However, cancer therapies often encounter limiting factors such as high toxicities and side effects. Here, we combined two anti-angiogenic epitopes that act on different pathways of angiogenesis into a single non-toxic cyclic peptide framework, namely MCoTI-II (*Momordica cochinchinensis* trypsin inhibitor-II), and subsequently assessed the anti-angiogenic activity of the novel compound. We hypothesized that the combination of these two epitopes would elicit a synergistic effect by targeting different angiogenesis pathways and result in improved potency, compared to that of a single epitope. This novel approach has resulted in the development of a potent, non-toxic, stable and cyclic analogue with nanomolar potency inhibition in *in vitro* endothelial cell migration and *in vivo* chorioallantoic membrane angiogenesis assays. This is the first report to use the MCoTI-II framework to develop a 2-in-1 anti-angiogenic peptide, which has the potential to be used as a form of combination therapy for targeting a wide range of cancers.

Over the past decade angiogenesis inhibitors have been a primary focus for cancer researchers, and understanding the various pathways of angiogenesis is vital for the design and development of next-generation therapies^1^. Angiogenesis inhibitor drugs currently on the market include the antibody bevacizumab (AVASTIN), and the small molecule drugs sorafenib (NEXAVAR) and sunitinib (SUTENT)^2^. Although they have been widely used in chemotherapy for the treatment of various cancers, there are drawbacks to their use – both alone and in combination therapies. In some cases these therapies can result in severe side effects, such as bleeding and clotting in arteries, which potentially lead to stroke or heart attack and hypertension^3^. Improvement of drug therapies is especially important for patients who are “high-risk” for surgical procedures, and those who may have complications near large blood vessels or other critical locations in the body^4^,^5^.

Peptides are an alternative class of molecules that have the potential to avoid some of the detrimental side effects of small-molecule drugs or antibodies, and the use and development of peptide-based therapeutics for cancer treatment is of particular interest in the pharmaceutical industry. The potential advantages of peptide-based therapies include lower immunogenicity than antibodies, and increased specificity towards the target of interest compared to small molecules^6^,^7^. To date, cilengitide is the only peptide-based anti-angiogenic drug that has entered clinical trials^8^. More generally, only limited numbers of peptide-based drugs have reached the pharmaceutical market, as peptides tend to have lower stability than small molecule drugs and are subject to proteolysis. However, this limitation could potentially be overcome by cyclic disulfide-rich peptides^9^,^10^,^11^.

Cyclic disulfide-rich peptides are a group of intermediate-sized molecules with the potential to overcome some of the stability limitations of current biopharmaceutical drugs. Side effects associated with small molecules might also be avoided through the higher target specificity of cyclic disulfide-rich peptides^9^,^12^. Some of the naturally occurring cyclic disulfide-rich peptides include kalata B1 (kB1)^13^, *Momordica cochinchinensis* trypsin inhibitor-II (MCoTI-II)^10^, and sunflower trypsin inhibitor-1 (SFTI-1)^11^. These peptides have high thermal and enzymatic stability; for peptides such as kB1 and MCoTI-II, this stability is due to the presence of the cyclic cystine knot (CCK), whereas for SFTI-1, stability results from the cyclic backbone and an extensive hydrogen-bonding network^11^.

The potential of these cyclic disulfide-rich peptides in pharmaceutical applications has recently been highlighted by the successful introduction of biologically active sequences into native cyclic peptide frameworks^14^ – a concept commonly known as ‘grafting’. The CCK framework contains six inter-cysteine loops and the SFTI-1 framework has two loops. All of these loops can potentially be used for epitope insertion, but the variation in loop size and structure means some epitopes are more appropriate for particular loops than others. The concept of grafting a single epitope to a specific target has been previously demonstrated in kB1, MCoTI-I, MCoTI-II and SFTI-1 frameworks^15^,^16^ using a range of therapeutic epitopes, including a bradykinin B_1_ antagonist^17^, pro-angiogenic sequences^18^, and a Hdm2/HdmX antagonist^19^. These grafted peptides have enhanced stability compared to their linear counterparts, and the ability to suppress unwanted activities, such as hemolytic activity. In addition, a previous study on the anti-angiogenic epitope polyR showed inhibitory activity against vascular endothelial growth factor A (VEGF-A) when grafted into the kB1 framework^20^. We have further examined the versatility of the polyR epitope in other cyclic disulfide-rich frameworks.

Multi-targeted therapy is a new paradigm for developing the next generation of cancer therapeutics, which emerged because conventional single-targeted therapies often encounter drug resistance issues^21^. To address this issue we have grafted anti-angiogenic epitopes into different loops of cyclic disulfide-rich peptide frameworks to enable the design of potent dual-targeting angiogenesis inhibitors. The concept of designing dual-targeting angiogenesis inhibitors is illustrated in Fig. 1. The anti-angiogenic epitopes chosen for this study included: *β*-turn-derived peptides from somatostatin (SST-01^22^ and SST-02^23^), which target the somatostatin receptor specific to neuroendocrine tumors; a pigment epithelium-derived factor (PEDF)^24^,^25^,^26^, which specifically targets the PEDF receptor for anti-inflammatory skin disorders and suppresses VEGF endothelial proliferation; and, an anti-VEGF-derived peptide from phage display (polyR), which specifically inhibits the interaction of VEGF with the kinase domain receptor (KDR, VEGF-R2)^27^,^28^,^29^. These epitopes have been shown to inhibit cell proliferation, cell migration and tumor growth in *in vitro* and *in vivo* models with low micromolar and nanomolar inhibition^22^,^23^,^24^,^25^,^27^,^28^. Overall, this study resulted in the development of a promising dual-targeting angiogenesis inhibitor and proved the feasibility of using cyclic disulfide-rich frameworks for multiple loop grafting, which augurs well for the future use of these frameworks in designing peptide-based combination drug therapies for cancer patients.

## Results

### Design and characterization of “first-generation” SFTI-1 and MCoTI-II grafted peptides

**Figure 1:**
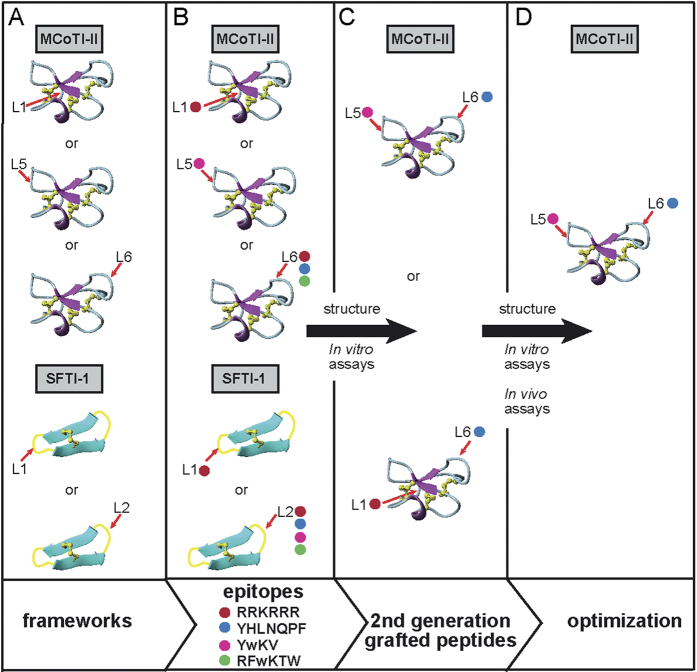
An overview of the screening process for the development of dual-targeting anti-angiogenic cyclic peptides, with a focus on the MCoTI-II framework. (**A**) Several potent anti-angiogenic sequences (pigment epithelium-derived factor (PEDF), somatostatin (SST) and polyR) were grafted onto SFTI-1 (PDB ID: 1JBL) and MCoTI-II (PDB ID: 1HA9) for initial *in vitro* bioactivity screening and NMR characterization. The PEDF sequence comprises residues Tyr 388 to Phe 394 from a human PEDF protein (Uniprot ID: P36955), two somatostatin mimetic sequences (SST-01 and SST-02) were derived from a human somatostatin receptor comprising residues Phe 109 to Thr 112, and an anti-VEGF mimetic (polyR) was derived from a phage display library^27^. The first tryptophan in the somatostatin epitopes was designed with a d-amino acid conformation, as a previous study showed this change is vital for its *β*-turn formation to selectively target SST2 and SST5 receptors^34^. (**B**) Anti-angiogenic epitopes that gave potent activity among these first-generation cyclic peptides were selected, and further grafted into the MCoTI-II framework. (**C**) The second-generation peptides then underwent similar screening as the first-generation peptides. (**D**) Only the best analogues, along with corresponding single-targeting counterparts, were tested in *in vivo* assays and further characterized by NMR. The location of epitope insertion into SFTI-1 and MCoTI-II are indicated with red arrows. Different epitope insertions into loops of the SFTI-1 and MCoTI-II frameworks are showed using different colored circles (i.e. red (represents polyR), blue (represents PEDF), pink (represents SST-01), and green (represents SST-02)).

**Figure 2:**
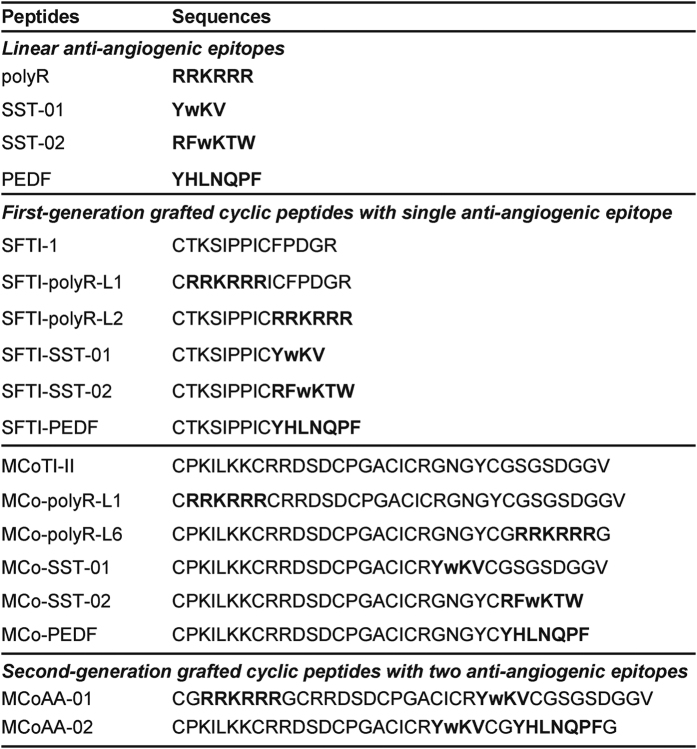
Sequences of grafted peptides with corresponding epitopes and native cyclic peptide frameworks. First-generation grafted cyclic peptides were designed with a single anti-angiogenic epitope inserted into a single loop. For the second-generation grafted cyclic peptides, two anti-angiogenic epitopes were inserted into two separate loops. Chosen anti-angiogenic epitopes are highlighted in bold. d-tryptophan is represented by ‘w’.

Small linear epitopes with potent anti-angiogenic activity were grafted into the MCoTI-II and SFTI-1 frameworks, with the aim of enhancing the stability of the epitopes while maintaining or improving potency. All anti-angiogenic epitopes chosen for this study were derived from a range of human proteins (Fig. 1). The loops chosen for grafting in SFTI-1 and MCoTI-II were based on previous successful examples^18^; in some cases, additional loops were used to explore structure–function relationships. A summary of the sequences of the epitopes, the native cyclic peptides, and the grafted peptides is given in Fig. 2.

### Structural analysis using NMR spectroscopy

**Figure 3:**
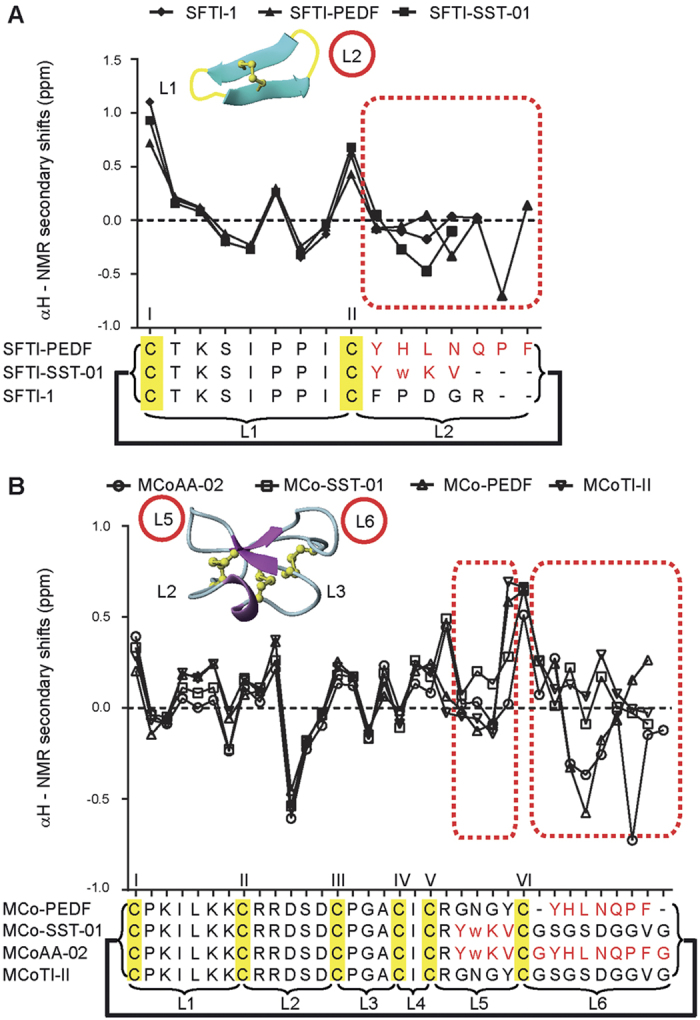
Structural analysis using NMR. (**A**) Comparison of SFTI-1 αH secondary shifts. (**B**) Comparison of MCoTI-II αH secondary shifts. All 2D NMR spectra were recorded at 298 K. All anti-angiogenic sequences are written in red and the regions for comparing native and grafted sequence are outlined by red dotted boxes. Disulfide bond connectivity is highlighted in yellow, and bold lines are used represent the cyclic nature of the peptides. Each cysteine is labeled with a Roman numeral and each loop is represented with the letter ‘L’. The loop of insertion of an anti-angiogenic sequence is circled in red for both SFTI-1 and MCoTI-II structures. All spectra were assigned using CCPNMR^46^ and each of the amino acid spin systems were specifically assigned based on Wuthrich *et al*.^47^. The αH secondary shifts were analyzed by subtracting the random coil ^1^H NMR chemical shifts of Wishart *et al*.^48^ from the experimental αH chemical shifts. The 3D molecular structure of SFTI-1 and MCoTI-II were illustrated using MOLMOL^49^.

One-dimensional spectra were recorded on all of the grafted peptides and showed well dispersed peaks in the amide region (Supplementary Figs S1 and S2) consistent with β-sheet containing peptides. Analysis of the secondary chemical shifts indicates that the grafted cyclic peptides have similar secondary structure to the native peptides. For example, the similarity in the secondary shifts of SFTI-SST-01 and SFTI-PEDF with the secondary shifts of native SFTI-1 indicates that both grafted cyclic peptides have similar structures to their native framework at all conserved residues, except at the locations of the grafted anti-angiogenic epitope (Fig. 3A). This conservation of native structure was also observed in grafted cyclic peptides with the MCoTI-II framework (Fig. 3B). Although the same epitopes were inserted into different cyclic frameworks, a difference in the secondary shifts was observed and can most probably be attributed to the different structural constraints or flexibility in the loops where the anti-angiogenic epitopes were inserted. The proline residues in the grafted peptides had the same conformations as the native cyclic frameworks (SFTI-1 Pro 6-*cis*, Pro 7*-trans*; MCoTI-II Pro 2-*trans* and Pro 15-*trans*) based on analysis of NOEs. The *trans* proline residues displayed strong NOEs between the δH’s of the proline residues and the αH of the preceding residue, whereas the *cis* proline residue displayed a strong NOE between the αH of the proline and the αH of the preceding residue. In addition, the proline residue in PEDF adopted a *trans* conformation when inserted into both SFTI-1 and MCoTI-II frameworks. A chemical shifts table of MCoAA-02 is included as Supplementary Table S2 and deposited in the Biological Magnetic Resonance Data Bank with the accession number 26882 (http://www.bmrb.wisc.edu/). The two-dimensional TOCSY and NOESY spectra of MCoAA-02 are shown in Supplementary Fig. S3.

### Linear epitopes, native and grafted cyclic peptides are non-toxic in a range of human cells

**Figure 4:**
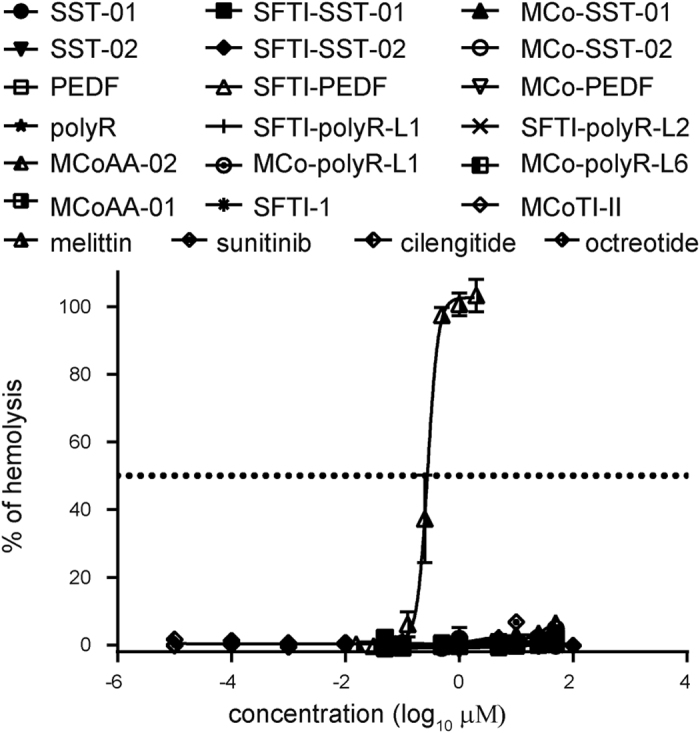
Hemolytic assay. Comparison of percentage hemolysis against human red blood cells for all peptides. Melittin was used as a positive control with 100% hemolysis. Drug controls (cilengitide, octreotide, and sunitinib) were also included in the assay. All data are shown as mean ± SD (n = 3).

All peptides and drug controls (cilengitide, octreotide, and sunitinib) were screened in both hemolytic (Fig. 4) and cell cytotoxicity assays (a 2-h incubation; data not shown), and the grafted peptides were found to be non-toxic to mammalian and human red blood cells.

### Grafted cyclic peptides showed specificity in inhibiting cell proliferation of HUVECs and cancer cells

Cell proliferation is one of the major processes to occur during tumor angiogenesis prior to cell migration^21^; thus, a reduction of cell proliferation during this time should inhibit new vascular growth to nearby tumor cells by stopping oxygen and nutrient supply. We determined the cell proliferation inhibitory activities of the linear epitopes, native and grafted cyclic peptides, and drug controls on HUVECs, MCF-7, PC-3, and HT-29 cells (Table 1). Cilengitide showed good potency in inhibiting the proliferation of HUVECs, consistent with this cell line previously being shown to have high levels of expression of integrins^30^. Octreotide was included in the study due to a previous report on its potent anti-angiogenic activity on HUVECs at concentrations within the range of 10^−10^–10^−6^ M; however, it had no effect on the proliferation of the cell lines tested in this study^31^. Full dose-response curves for all peptides listed in Table 1 are included in Supplementary Fig. S4. Neither of the native cyclic frameworks (SFTI-1 and MCoTI-II) inhibited cell proliferation in any of the cell lines at concentrations >100 μM. The linear epitopes and grafted peptides had varying degrees of inhibitory activity, likely due to the different types of receptors expressed within each cell line (the greater the number of types, the greater the number of potential targets), or the level at which the receptors are expressed (affecting the number of possible binding sites)^30^,^31^. Several grafted cyclic peptides showed better inhibition of proliferation compared to their linear counterparts, potentially due to the greater stability conferred upon grafting into a cyclic framework. However, there are also examples where the grafted cyclic peptides had less inhibition on some cell lines in the proliferation assay compared to the linear form. It is possible the small peptides are restrained in the cyclic peptides in such a way as to inhibit binding to the appropriate target (Table 1).

### Screening of peptides in VEGF-mediated HUVEC migration assay

**Figure 5:**
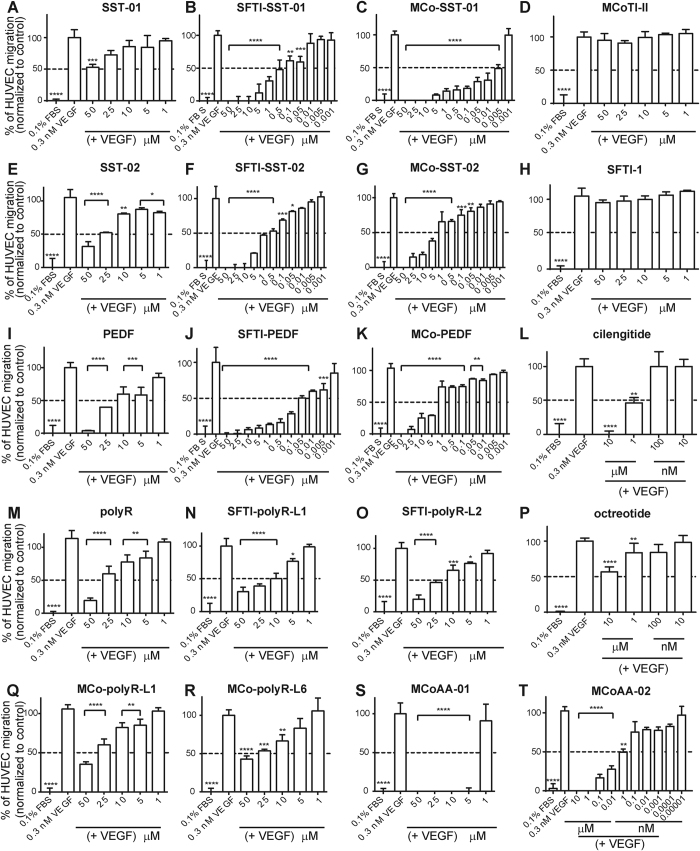
Cell migration on VEGF-mediated HUVECs. The percentage of HUVEC migration was analyzed using Prism Version 6 (GraphPad). (**A–T**) VEGF was used as a positive control with 100% HUVEC migration. All peptides were tested at concentrations ranging from 0.001–50 μM except for cilengitide and octreotide, which were tested in 10-fold dilutions from 10 μM. Peptides were added in the presence of 0.3 nM VEGF (bottom chamber). Data are shown as mean ± SD (n ≥ 3). Data were normalized to the mean VEGF control. P-values are represented as follows: ****p ≤ 0.0001, ***p ≤ 0.001, **p ≤ 0.01, and *p ≤ 0.05. A one-way ANOVA with Dunnett’s post-test using a multiple comparison test was used for statistical analysis.

Although the grafted cyclic peptides were inhibitory in the cell proliferation study, the ability to inhibit cell migration also plays an important role in tumor angiogenesis. Consequently, all peptides, including cilengitide and octreotide, were tested in a VEGF-mediated HUVEC migration assay. VEGF was included as a positive control as it is a potent angiogenic growth factor that stimulates HUVEC migration (taken as 100% in the assay). All first-generation peptides showed better inhibition of HUVEC migration compared to their linear epitopes. Neither MCoTI-II nor SFTI-1 showed any effect on HUVEC migration and were subsequently treated as negative controls. The MCoTI-II grafted peptides containing the SST-01 and PEDF epitopes showed 50% inhibition on HUVEC migration at 0.005 μM and between 1 to 5 μM, respectively. The most potent SFTI-1 graft contained the PEDF epitope, which displayed 50% inhibition on HUVEC migration at 0.05 μM, as shown in Fig. 5.

### Design and characterization of dual-targeting grafted analogues

Second-generation analogues, containing two grafted epitopes, were designed based on the activity observed in cell proliferation and migration assays, as well as the NMR characterization studies. MCoTI-II was chosen as the second-generation framework for dual-epitope insertion because the larger number of loops and loops of different sizes offer greater scope for epitope insertion compared to SFTI-1. For the second-generation peptides (MCoAA-01 and MCoAA-02), the SST-01 epitope was grafted into loop 5, consistent with the potent first-generation peptide, and the polyR and PEDF epitopes were grafted into loop 1 and loop 6, respectively. The locations of the polyR and PEDF epitopes were primarily based on the first-generation cell migration assay results. The polyR and PEDF epitopes were flanked by glycine residues to assist in peptide folding. The addition of the glycine residues maintained the original loop size of the native cyclic framework and were unlikely to have steric clashes during the folding process. Figure 1 illustrates the stages in the development of a potent dual-targeting cyclic peptide. A full list of peptide molecular masses is shown in Supplementary Table S1. All peptides were purified to >95% before being used in the assays. An example of purity check and mass spectrometry data of MCoAA-02 is provided in Supplementary Fig. S5.

Both MCoAA-01 and MCoAA-02 had native-like structures based on NMR analysis. For MCoAA-02, the secondary shifts of the inserted anti-angiogenic epitope in loops 5 and 6 (Fig. 3B) were comparable to those grafted cyclic peptides with single anti-angiogenic epitopes (i.e., MCo-SST-01 and MCo-PEDF). Interestingly, MCoAA-01 and MCoAA-02 are capable of targeting particular cell lines with higher potency than peptides with a single epitope insertion. MCoAA-01 inhibited HUVEC proliferation with a higher potency than MCo-SST-01 and MCo-PEDF. Similarly, MCoAA-02 inhibited HT29 cell proliferation more potently than MCo-SST-01 and MCo-PEDF. However, there are exceptions in some cell lines where a single epitope insertion inhibited cell proliferation more potently compared to the dual-targeting peptides. These differences might be related to variable expression levels of targeted receptors on these cell lines. MCoAA-02 was observed to be the most potent second-generation dual-targeting cyclic peptide, with 50% inhibition of HUVEC migration at 1 nM (Fig. 5T); for cilengitide, the same degree of inhibition required a concentration of 1 μM (Fig. 5L). Images of the final step in the cell migration assay with MCoAA-02, prior to absorbance reading, are shown in Supplementary Fig. S7. Our data correlate well with that supplied by the manufacturer, with 10 μM of cilengitide resulting in 100% inhibition of HUVEC attachment on vitronectin and fibronectin. Sunitinib, a potent multi-targeted tyrosine kinase small molecule inhibitor^32^, was also tested at 10 μM and showed 100% inhibition of HUVEC migration (Supplementary Fig. S8A), which was similar to the effect of cilengitide and MCoAA-02 at this concentration. By contrast, octreotide showed a lower potency compared to cilengitide and MCoAA-02 between 1–10 μM.

### The effect of the potent dual-targeting cyclic peptide MCoAA-02 on blood vessel growth in the *in vivo* CAM model

**Figure 6:**
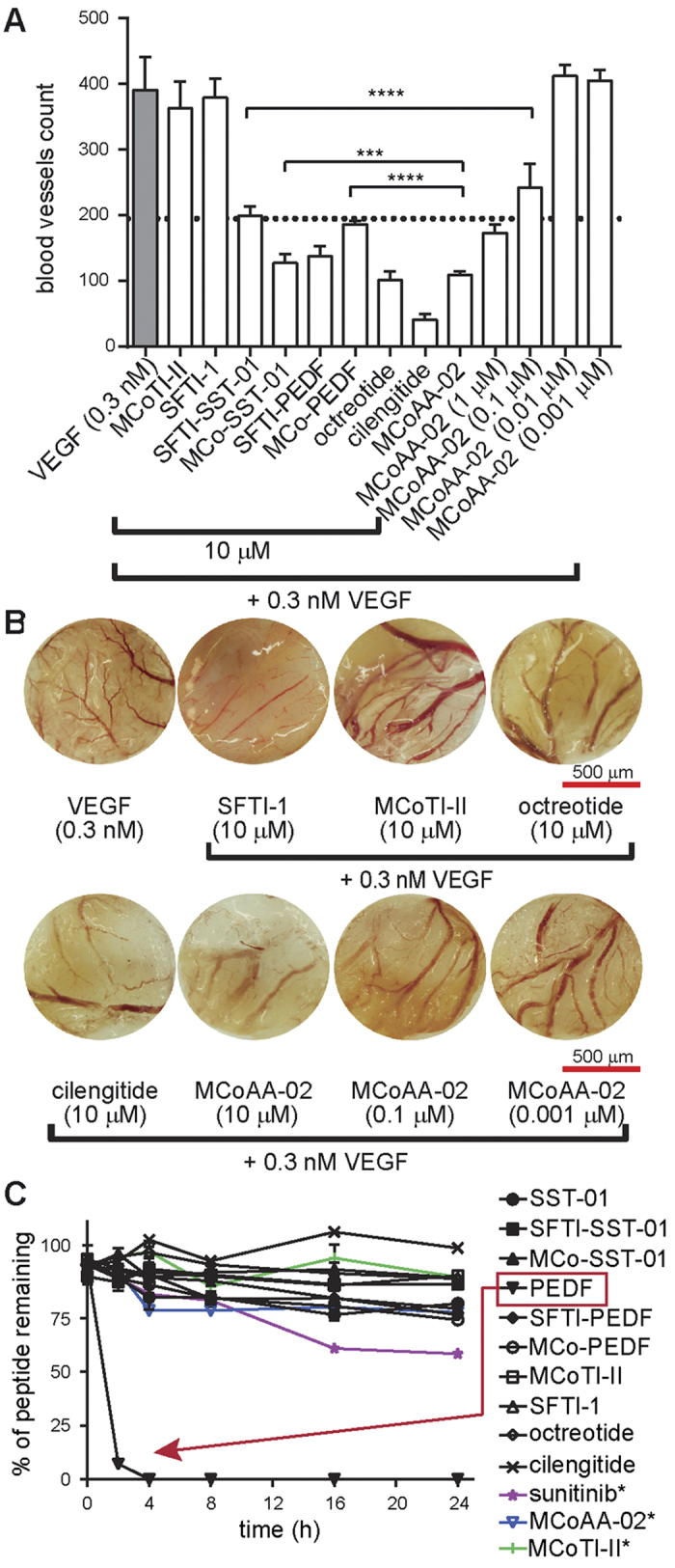
Chorionallantoic membrane and stability assays. (**A**) Comparison of blood vessel count of the linear and grafted peptides. Error bars indicate ± SD (n ≥ 6). The dotted line indicates ~50% inhibition of blood vessels. A one-way ANOVA with Dunnett’s post-test using a multiple comparison test was used for statistical analysis. In addition, unpaired t-test was used to test the significance of MCoAA-02 against MCo-SST-01 and MCo-PEDF. ****p ≤ 0.0001 and ***p ≤ 0.05. All peptides were compared to 0.3 nM VEGF (highlighted in grey), which is represented as 100% blood vessel growth. (**B**) This diagram shows the blood vessel growth of MCoAA-02 at various concentrations compared to the cyclic frameworks SFTI-1 and MCoTI-II in the CAM assay. VEGF was used as the positive control, and octreotide and cilengitide as the negative controls. All images were taken with an original magnification of x16 on an Olympus SZX12 dissecting microscope with a light box. DP capture and DP manager software packages were used during image acquisition. (**C**) This graph illustrates the percentage of peptide remaining over 24 h in the serum stability assay. All compounds showed better stability than the linear PEDF peptide (highlighted with red dashed lines). All data are represented as mean ± SD and were recorded in triplicate. Peptides labeled with an asterisk (*) were tested using the same method except with an additional step – dissolving the centrifuged pellet with 8 M guanidinium chloride before RP-HPLC analysis.

Based on the potency of MCoAA-02 in the cell assays, it was also tested in a CAM assay to determine the effect on blood vessel growth *in vivo* (Fig. 6A). Octreotide, cilengitide and selected grafted cyclic peptides were included for comparative purposes, and VEGF (0.3 nM) was used as a positive control. To evaluate the inhibitory effect of these peptides on blood vessel growth, VEGF was added simultaneously to all peptides during the assay. Peptides were tested at 10 μM, as most grafted cyclic peptides showed more than 50% inhibition in the cell migration assay at this concentration, with the exception of MCoAA-02, which was tested at concentrations ranging from 0.001 μM to 10 μM. MCoAA-02 more potently inhibited blood vessel growth compared to analogues with only single anti-angiogenic epitope insertions. An improvement in blood vessel growth inhibition, by approximately 42%, was observed for MCoAA-02 compared to MCo-PEDF. Although MCoAA-02 was not as potent as cilengitide in inhibiting blood vessel growth, it showed comparable results to octreotide at 10 μM. In addition, MCoAA-02 was capable of inhibiting approximately 50% of blood vessel growth at 100 nM (Fig. 6A). Sunitinib was also tested and found to be more potent than cilengitide and octreotide at 10 μM (Supplementary Fig. S8B,C). A comparison of excised CAM images of MCoAA-02 (0.001, 0.1 and 10 μM), cyclic frameworks (MCoTI-II and SFTI-1), cilengitide, octreotide, and VEGF is shown in Fig. 6B.

### Peptide stability assessment in human serum

The stability of peptides tested in the CAM assay was examined against human serum for 24 h. All peptides were stable when subjected to human serum except PEDF, a short linear peptide, which was degraded within 4 h, as expected. Interestingly, SST-01, which contains a d-amino acid, was found to be more stable than PEDF. The stability of MCoAA-02 was compared to sunitinib and MCoTI-II using the method described by Ji and colleagues^19^. Although MCoAA-02 was found to be slightly less stable than its native framework (MCoTI-II), more than 75% of this peptide remained in the serum over 24-h, which was approximately 20% higher than the percentage of sunitinib that remained after the same amount of time (Fig. 6C). Overall, results from this assay showed the native cyclic frameworks and all grafted cyclic peptides to be remarkably stable.

## Discussion

Peptide therapeutics are known to have excellent selectivity and efficacy, low toxicity, and lower production costs than antibodies; however, they are prone to many challenges, including proteolytic instability and short *in vivo* half-lives^33^. These challenges can potentially be overcome by a range of chemical modifications to improve their physicochemical properties^33^. Despite these approaches for improving stability, many FDA-approved drugs are either small molecules or antibodies in the anti-angiogenic field, with only two cyclic peptide-based drugs – cilengitide and octreotide – currently in late-stage clinical trials^2^ or on the market^34^, respectively. These developments suggest that peptide-based anti-angiogenic therapeutics is an area of potential growth.

One approach for improving stability of bioactive peptides is to conjugate them with more stable molecules. However, when short bioactive epitopes are conjugated externally on a molecule, they are more susceptible to degradation, as they are more readily accessible by enzymes and typically require further chemical modification to achieve acceptable pharmacokinetic properties^35^. Based on this limitation, our study aimed to explore how short anti-angiogenic epitopes can be cyclized within a single framework to improve stability and bioactivity. In addition, a dual-targeting strategy was also explored to examine the feasibility of incorporating two short anti-angiogenic epitopes into a single framework. As in the recent use of a dual-targeting strategy to target an epidermal growth factor receptor mutation in non-small-cell lung cancer patients by combining two individual drugs (i.e., afatinib and cetuximab), resulting in dramatic inhibition of tumor growth. Cetuximab alone showed significant suppression of tumors with the L858R mutation but only a slight response to tumors with the T790M mutation, highlighting the advantage of using the combination therapy^36^.

**Figure 7:**
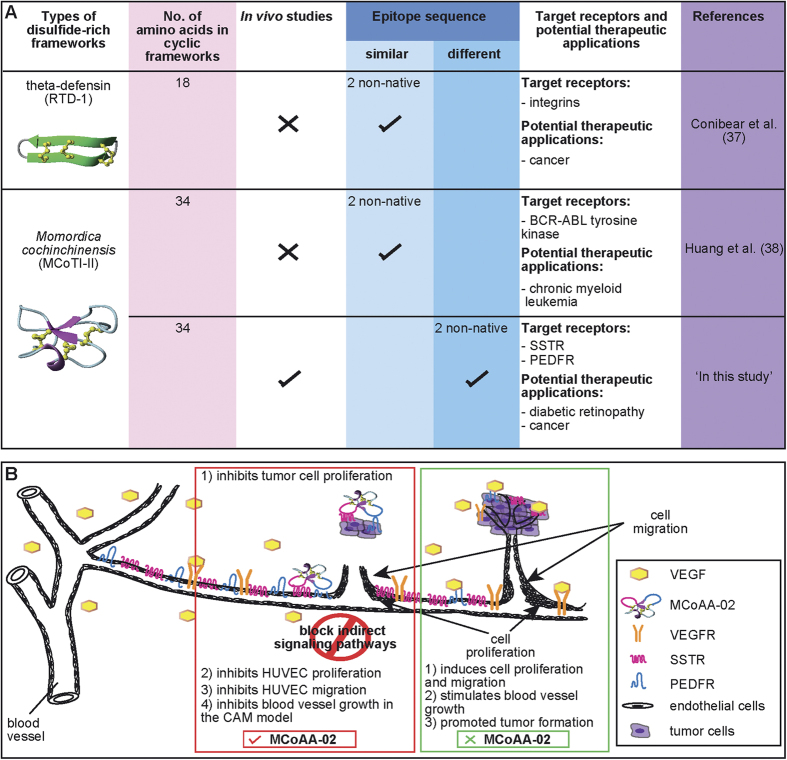
A comparison of recent work using cyclic disulfide-rich frameworks for bi-functional studies and a schematic overview of how MCoAA-02 could inhibit the angiogenic process. (**A**) A comparison of dual-targeting peptides; specifically, a comparison of the findings of this study to previous work using cyclic disulfide-rich peptide frameworks originating from plant and animal sources. Previous work focused only on grafting two identical epitopes. Our work is the first utilizing the MCoTI-II cyclic framework for the insertion of two different anti-angiogenic targets. (**B**) Schematic diagram depicting how MCoAA-02 has the potential to act as a dual-targeting cyclic peptide by blocking indirect signaling pathways to exert a synergistic anti-angiogenic effect.

Many new opportunities for the development of peptide therapeutics are likely to arise from multifunctional peptides, and this is an area that needs further exploration^33^. Limited studies have used the dual-targeting strategy with cyclic, disulfide-rich frameworks; indeed, there are only a few examples of bi-functional peptides using cyclic frameworks^37^,^38^. Previous examples in this area have demonstrated the insertion of two similar epitopes targeting an identical receptor, as summarized in Fig. 7A. Although an increase in activity was observed compared to their corresponding singly grafted peptides, they did not show comparable or better effects compared to drug controls. In the current study, we demonstrated the rational design of second-generation dual-targeting cyclic anti-angiogenic peptides with two different grafted epitopes. Both grafted peptides produced nanomolar potency in both *in vitro* and *in vivo* studies, and showed comparable effects to cyclic peptide drug controls and higher stability in human serum than the orally active sunitinib.

All peptides used in this study were non-toxic to either cancer or normal cells, which allowed further investigation on the bioactivity of these peptides in a range of assays. An increase in the inhibitory activity of cell migration studies was observed on grafted peptides when compared to the activity of their corresponding anti-angiogenic epitopes. This increase in potency is probably due to the increase in stability from grafting the epitopes into a cyclic framework. In this study, we have also demonstrated grafting epitopes into different locations of the same or different frameworks resulted in different biological effects. For example, differences in potency were observed for the polyR epitope when inserted into different loops of the same framework. This suggests that to gauge the best location of epitope insertion, a thorough understanding of the native framework structure is essential and screening using a range of assays to identify the location that results in the most significant increase in activity is important for the design of dual-targeting cyclic peptides. Among the grafted cyclic peptides, the dual-targeting MCoAA-01 and MCoAA-02 peptides were the most potent in the cell proliferation studies, with inhibition observed for several cell lines similar to the inhibitory effects in drug controls such as cilengitide and sunitinib. In particular, MCoAA-01 and MCoAA-02 most potently affected HUVECs and HT-29 cells, respectively. This can be explained by the expression of SST and PEDF receptors in these cell lines, both of which are required for these dual-targeting peptides^39^. When these peptides were examined in the cell migration assay, MCoAA-02 exhibited greater potency than MCoAA-01. At 50% inhibition of HUVEC migration, MCoAA-02 exerted 1000-fold higher potency than cilengitide; hence, it was further characterized in *in vivo* studies.

Given that MCoTI-II did not inhibit blood vessel growth in the CAM assay, it appears the anti-angiogenic activity of MCoAA-02 is derived solely from the grafted epitopes and not the cyclic framework. Furthermore, MCoAA-02 was more active in the CAM assay than MCo-SST-01 or MCo-PEDF, suggesting the inhibitory effect of blood vessels growth on MCoAA-02 could either be due to an additive or synergistic effect. More studies will be required in future to determine the specific effect of this peptide. Angiogenesis inhibition and tumor growth regulation events are both indirect effects of somatostatin. Therefore, MCoAA-02 could potentially have an indirect effect in regulating VEGF on endothelial or tumor cells by inhibiting signaling events downstream of the SST and PEDF receptors, which results in the suppression of cell proliferation and migration. This hypothesis is supported by studies by Craword^40^ and Rai and colleagues^41^, who suggested that the anti-angiogenic effect caused by PEDF could be due to the suppression of pro-angiogenic miRNAs (e.g., miR-126, miR-23b and miR-27b). They suggest that blocking of these miRNAs leads to inhibition of endothelial cell proliferation, and the blocking of the SST receptor activates downstream signaling cascades such as MAPK or ERK pathways, and protein tyrosine phosphatases, which could lead to cell cycle arrest.

A schematic diagram outlining our proposed mechanism of action of MCoAA-02 on endothelial and tumor cells is shown in Fig. 7B. We propose that MCoAA-02 could inhibit cell proliferation of cancer and endothelial cells, followed by the inhibition of endothelial cell migration to nearby tumor cells and ultimately block nutrient supply. Together, these events could result in the inhibition of blood vessel growth, consistent with the data from the CAM assay. Furthermore, MCoAA-02 could indirectly regulate VEGF on endothelial or tumor cells by inhibiting signaling events downstream of the SST and PEDF receptors, resulting in the suppression of cell proliferation, migration and blood vessel growth. In contrast, in the absence of MCoAA-02 during an angiogenesis event, VEGF acts as a potent angiogenesis growth factor by binding to its corresponding receptor, VEGFR and stimulating cell proliferation, migration and blood vessel growth, as shown in Fig. 7B.

Apart from the biological activities of these peptides, peptide stability is also an important factor when designing new peptide therapeutics, as it can enhance the probability of success in pharmacokinetic studies. Despite the significant number of residue changes in MCoAA-02 compared to MCoTI-II, both are stable in human serum over 24-h. This similarity in stability is presumably related to the similarities in structure reflected in the chemical shifts. Interestingly, MCoAA-02 was more stable in human serum than sunitinib. The stability of MCoAA-02 combined with its lack of toxicity to normal or cancerous cells highlights its potential as a candidate for the design of dual- or multi-targeting peptide therapeutics targeting cancer applications. PEDF and SST-01 epitopes have also been implicated in treating ocular vascular diseases such as diabetic retinopathy^40^,^41^,^42^,^43^, suggesting that MCoAA-02 might also have applications in this field.

Although there are examples of combinatory approaches based on somatostatin, which include the conjugation of somatostatin to different chemotherapy drugs such as camptothecin and methotrexate, resulting in improved efficacy with fewer side effects^39^, this is the first study combining a somatostatin epitope with another anti-angiogenic epitope in a single cyclic disulfide-rich framework. This study thus potentially opens up new possibilities of using cyclic disulfide-rich frameworks as an alternative strategy for the development of next-generation peptide-based combination therapies. A further understanding of the specific mechanism of action on endothelial and cancer cells, along with *in vivo* mouse model studies, could also enhance its relevance in a clinical setting. In addition, applying this strategy to other cyclic frameworks could lead to applications in a wide range of human diseases.

## Materials and Methods

### Peptide synthesis

Grafted peptides (all peptides listed in Table 1 except linear anti-angiogenic epitopes and MCoTI-II) and SFTI-1 were synthesized using solid-phase peptide synthesis on a CS Bio synthesizer using Boc chemistry. Peptides were constructed on Boc-Gly-PAM resin (Chem-Impex International) with S-tritylmercaptopropionic acid (Peptides International) as a linker using *N,N,N′,N′*-tetramethyl-*O*-(6-chloro-1H-benzotriazol-1-yl)uronium hexafluorophosphate (HCTU) for amino acid activation. Linear peptide analogues (polyR, PEDF, SST-01, SST-02) were synthesized using Fmoc chemistry on a Symphony microwave synthesizer using chlorotrityl chloride resin. Peptides made using Boc chemistry were cleaved from the resin using hydrogen fluoride with *p*-cresol as a scavenger at 0 to 5 °C for 1 h. Fmoc-synthesized peptides were cleaved using a mixture of 95% trifluoroacetic acid (TFA)/2.5% triisopropylsilane/2.5% H_2_O. The TFA was removed by rotary evaporation and the residue partitioned between 50% acetonitrile in water containing 0.1% TFA and cold diethyl ether. All grafted peptides and linear peptides were synthesized on a 0.5 mmol scale. The aqueous layer was lyophilized and the resulting crude peptides were purified using reverse-phase HPLC (RP-HPLC). MCoTI-II was isolated from *M. cochinchinensis* seed extract, as described by Chan *et al*.^44^. Cilengitide (EMD 121974; catalog no. S7077; MW: 588.66) and octreotide acetate (SANDOSTATIN; catalog no. P1017; MW: 1019.28) were purchased from Selleck Chemicals. Both drugs are cyclic peptide mimetics. Sunitinib malate, a small molecule multi-targeted receptor tyrosine inhibitor, was purchased from LC laboratories (SUTENT; catalog no. S-8803; M.W: 532.56).

### Peptide purification and folding conditions

Crude peptides obtained from either plant extracts or chemical synthesis were purified using a series of Phenomenex C18 columns on RP-HPLC. Gradients of 1%/min of 0–80% solvent B (90% MeCN in 0.045% TFA in H_2_O) and solvent A (aqueous 0.05% TFA in H_2_O) were used and the eluent was monitored at 215 and 280 nm. Peptide purity was examined on a Nexera UHPLC (Shimadzu) with a flowrate of 0.4 mL/min on a 0.8 mL/min Agilent column using a 2% gradient of 0–50% solvent B and masses were determined by electrospray mass spectrometry. All peptides used in assays had >95% purity. Folding trials were carried out using 1 mg/mL aliquots of peptide in various folding buffers of different pHs, as well as different concentrations of ammonium bicarbonate and dimethyl sulfoxide (DMSO). The optimal conditions for peptide folding were as follows: SFTI-1 grafted peptides were folded in two steps: cyclization with 0.1 M ammonium bicarbonate (pH 8.0)/0.1 M TCEP, then oxidation in 0.1 M ammonium bicarbonate (pH 8.0); MCoTI-II grafted peptides were folded in a one-pot oxidation solution of 0.1 M ammonium bicarbonate (pH 8.5) except for MCo-SST-01, MCo-SST-02, MCoAA-01 and MCoAA-02, which required the addition of 10% DMSO. Examples of LC-MS traces of folded MCoAA-01 and MCoAA-02 with the above conditions are provided in Supplementary Fig. S6.

### Cell culture

HUVECs (human umbilical vein endothelial cells) were cultured in EGM™-2 BulletKit™ supplemented with SingleQuots™ (supplements: growth factors, cytokines, antibiotics; Lonza) and 10% FBS (catalog no. FFBS-500; Scientifix). Both HT-29 and MCF-7 cells were cultured in 10% FBS/DMEM (Dulbecco’s Modified Eagle Medium) with 1% penicillin–streptomycin (5000 U/mL; Life Technologies), whereas PC3 cells (prostate cancer cells) were cultured in 10% FBS/RPMI (Roswell Park Memorial Institute) 1640 with 1% penicillin–streptomycin (5000 U/mL; Life Technologies). All cells were maintained at 37 °C in 5% CO_2_.

### Cell cytotoxicity and proliferation assays on HUVECs (non-cancerous) and cancerous cells

Cell cytotoxicity and proliferation assays were performed using similar methods to those described by Chan *et al*.^45^. All cells were maintained using the media conditions described above, and passages two to ten were used for all cell lines. For all cell lines, 5.0 × 10^3^ cells/well (100 μL) and 1.5 × 10^3^ cells/well (100 μL) were used for cell cytotoxicity and cell proliferation assays, respectively. All peptides (10 μL, at final concentrations ranging from 0.05–100 μM) and drugs, which included cilengitide, octreotide and sunitinib (at final concentrations ranging from 0.00001–100 μM), were used in both assays. Triton X-100 (0.1% (v/v); 10 μL) was used as a positive control. Cells were treated with fresh media the day after plating, before the addition of peptides, followed by a subsequent 2-h and 48-h incubation for cell cytotoxicity and cell proliferation, respectively. After different incubation periods, 3-(4, 5-dimethylthiazolyl-2)-2,5-diphenyltetrazolium bromide (MTT) (10 μL; 5 mg/mL in PBS) was added, and cells were incubated for a further 4 h. The supernatant was then removed and 100 μL DMSO added to solubilize formazan salts. Experiments were performed in triplicate. Cell numbers were recorded at 600 nm. All data represent average mean ± SD.

### Transwell migration assay using HUVECs

A cell migration assay was performed as described by Chan *et al*.^45^ HUVECs between passages three and ten were used. Briefly, initial peptide concentrations of 10–500 μM were prepared for all peptides except SFTI-SST-01, MCo-SST-01, SFTI-SST-02, MCo-SST-02, SFTI-PEDF, MCo-PEDF (0.01–500 μM), cilengitide, octreotide, and MCoAA-02 (1 × 10^−7^ M–100 μM). To the apical well, 100 μL of 1 × 10^5^ cells/well were seeded and 600 μL of medium supplemented with or without 0.3 nM VEGF (Sigma, catalogue no. V7259) was added to the basolateral well. The well containing cells with 0.1% FBS/EGM-2 basal medium was used as a negative control, and the well containing cells with 0.3 nM VEGF was used as a positive control. All peptides were stimulated with 0.3 nM VEGF. Experiments were done in triplicate. All data represent mean ± SD. Prism Version 6 software (GraphPad) was used for statistical analysis. A one-way analysis of variance (ANOVA) with Dunnett’s multiple comparison test was applied in the analysis.

### Hemolytic assay

Human red blood cells were used to assess the toxicity of the peptides. Peptides and melittin were tested at concentrations ranging from 0.05–50 μM and 0.015–2 μM, respectively. Cilengitide, octreotide and sunitinib were tested at 10-fold dilutions from 0.00001–10 μM. These experiments were performed as described previously^44^.

### Peptide stability assay

The overall procedures used to assess peptide stability are based on those described previously by Chan *et al*.^18^ and Ji *et al*.^19^. A peptide concentration of 300 μM was used, and time points taken at 0, 2, 4, 8, 16, and 24 h. The stability of sunitinib was also examined using this method, except an additional step was added after the final centrifugation step, whereby the pellet was dissolved in 8 M guanidine hydrochloride (300 μL) prior to analysis with RP-HPLC; this additional step was adapted from Ji *et al*.^19^. Supernantants were analyzed on an Agilent RP-HPLC using a linear 1%/min gradient of 0–50% solvent B with a 0.3 mL/min Phenomenex column. Peak height was used for integration of data at 215 nm and each experiment was performed in triplicate.

### Inhibition of blood vessel growth using an *in vivo* CAM model

The chorioallantoic membrane (CAM) assay was performed as described by Chan *et al*.^18^. Briefly, fertilized quail (*Cortunix cortunix*) eggs were purchased from a local supplier (Jimboomba, Queensland, Australia) and incubated at 37 °C for 4 days in a humidified incubator prior to the start of the assay. All peptides were tested at 10 μM, except MCoAA-02, which was tested at concentrations ranging from 0.001 μM to 10 μM with the addition of VEGF (final concentration: 0.3 nM). VEGF solution and DMEM were used as positive and negative controls, respectively. Images were taken on an Olympus SZX12 dissecting microscope with an original magnification of x16. DP capture and DP manager software packages were used during image acquisition. Blood vessel number was quantified by eye, using a hand tally counter. A one-way ANOVA was applied for the statistical comparison analysis using Prism Version 6 software (GraphPad). Each test peptide was compared with the positive control, and results were represented as mean blood vessel count. *P* < 0.05 was considered significant. All data represents average mean ± SD (n ≥ 6).

### NMR spectroscopy

Samples were dissolved in 90% H_2_O/10% D_2_O (D_2_O; 99.9% purity; Cambridge Isotope Laboratories, Woburn, MA) with a final concentration of 1 mM at pH 5.5. 4,4-Dimethyl-4-silapentane-1-sulfonic acid (DSS) was used as a chemical shift reference for spectral calibration. One-dimensional (1H) and two-dimensional (TOCSY and NOESY) spectra were recorded for all native and grafted peptides at 298 K on a Bruker Avance 600 MHz spectrometer, with mixing times of 200–300 ms for NOESY experiments. All spectra were assigned using CCPNMR^46^. All amino acid spin systems were specifically assigned based on Wuthrich *et al*.^47^. For the αH secondary shifts, these were analyzed based on subtracting the random coil ^1^H NMR chemical shifts of Wishart *et al*.^48^ from experimental αH chemical shifts. The three-dimensional molecular structure of SFTI-1 and MCoTI-II were illustrated using MOLMOL^49^.

### Statistical analysis

Results are expressed as mean ± standard deviation (SD) of the mean. Statistical significance was evaluated with one-way ANOVA followed by Dunnett’s multiple comparison test, using GraphPad Prism Version 6 software.

## Additional Information

**How to cite this article**: Chan, L. Y. *et al*. Dual-targeting anti-angiogenic cyclic peptides as potential drug leads for cancer therapy. *Sci. Rep.* **6**, 35347; doi: 10.1038/srep35347 (2016).

## Supplementary Material

Supplementary Information

## Figures and Tables

**Table 1:** Cell proliferation inhibitory activity of all grafted and native peptides on HUVECs and cancer cells.

Peptides	IC_50_ (μΜ) ± S.D.; 48 hours			
	HUVEC	MCF-7	PC3	HT-29
SST-01	57.54 ± 0.04	>100	>100	>100
SFTI-SST-01	18.32 ± 0.05	46.07 ± 0.05	>100	45.38 ± 0.03
MCo-SST-01	17.12 ± 0.05	>100	85.44 ± 0.08	74.13 ± 0.07
SST-02	>100	>100	>100	>100
SFTI-SST-02	>100	55.13 ± 0.07	>100	43.29 ± 0.04
MCo-SST-02	>100	70.61 ± 0.11	83.55 ± 0.20	58.89 ± 0.09
PEDF	19.40 ± 0.08	>100	>100	>100
SFTI-PEDF	27.89 ± 0.11	>100	>100	>100
MCo-PEDF	50.16 ± 0.15	53.44 ± 0.14	39.11 ± 0.07	35.22 ± 0.08
polyR	44.64 ± 0.03	83.51 ± 0.46	>100	>100
SFTI-polyR-L1	>100	28.72 ± 0.15	>100	82.27 ± 0.13
SFTI-polyR-L2	>100	41.84 ± 0.07	>100	>100
MCo-polyR-L1	>100	>100	>100	>100
MCo-polyR-L6	34.95 ± 0.03	>100	>100	52.89 ± 0.11
MCoAA-01	10.83 ± 0.09	45.01 ± 0.16	>100	40.12 ± 0.13
MCoAA-02	27.06 ± 0.06	>100	35.44 ± 0.13	19.75 ± 0.07
SFTI-1	>100	>100	>100	>100
MCoTI-II	>100	>100	>100	>100
cilengitide	4.69 ± 0.06	>100	>100	84.68 ± 0.13
octreotide	>100	>100	>100	>100
sunitinib	19.10 ± 0.46	17.07 ± 0.11	16.24 ± 0.26	1.43 ± 0.24

## References

[b1] Carmeliet P. & Jain R. K. Angiogenesis in cancer and other diseases. Nature 407, 249–257 (2000). 11001068 10.1038/35025220

[b2] Singh M. & Ferrara N. Modeling and predicting clinical efficacy for drugs targeting the tumor milieu. Nat. Biotechnol. 30, 648–657 (2012). 22781694 10.1038/nbt.2286

[b3] Shojaei F. Anti-angiogenesis therapy in cancer: current challenges and future perspectives. Cancer Lett. 320, 130–137 (2012). 22425960 10.1016/j.canlet.2012.03.008

[b4] Thornton A. D., Ravn P., Winslet M. & Chester K. Angiogenesis inhibition with bevacizumab and the surgical management of colorectal cancer. Br. J. Surg. 93, 1456–1463 (2006). 17115389 10.1002/bjs.5624

[b5] Chua T. C. Gastrointestinal oncological surgery in patients with metastatic cancer treated with angiogenesis inhibitors: safe or not? ANZ J. Surg. 79, 672–673 (2009). 19878155 10.1111/j.1445-2197.2009.05047.x

[b6] Otvos L. Jr. & Wade J. D. Current challenges in peptide-based drug discovery. Front. Chem. 2, 62 (2014). 25152873 10.3389/fchem.2014.00062 PMC4126357

[b7] Craik D. J., Fairlie D. P., Liras S. & Price D. The future of peptide-based drugs. Chem. Biol. Drug Des. 81, 136–147 (2013). 23253135 10.1111/cbdd.12055

[b8] Reynolds A. R. . Stimulation of tumor growth and angiogenesis by low concentrations of RGD-mimetic integrin inhibitors. Nat. Med. 15, 392–400 (2009). 19305413 10.1038/nm.1941

[b9] Craik D. J., Daly N. L., Bond T. & Waine C. Plant cyclotides: a unique family of cyclic and knotted proteins that defines the cyclic cystine knot structural motif. J. Mol. Biol. 294, 1327–1336 (1999). 10600388 10.1006/jmbi.1999.3383

[b10] Hernandez J. F. . Squash trypsin inhibitors from Momordica cochinchinensis exhibit an atypical macrocyclic structure. Biochemistry 39, 5722–5730 (2000). 10801322 10.1021/bi9929756

[b11] Luckett S. . High-resolution structure of a potent, cyclic proteinase inhibitor from sunflower seeds. J. Mol. Biol. 290, 525–533 (1999). 10390350 10.1006/jmbi.1999.2891

[b12] Craik D. J. Joseph Rudinger memorial lecture: discovery and applications of cyclotides. J. Pept. Sci. 19, 393–407 (2013). 23737440 10.1002/psc.2523

[b13] Saether O. . Elucidation of the primary and three-dimensional structure of the uterotonic polypeptide kalata B1. Biochemistry 34, 4147–4158 (1995). 7703226 10.1021/bi00013a002

[b14] Clark R. J., Daly N. L. & Craik D. J. Structural plasticity of the cyclic-cystine-knot framework: implications for biological activity and drug design. Biochem. J. 394, 85–93 (2006). 16300479 10.1042/BJ20051691 PMC1386006

[b15] Poth A. G., Chan L. Y. & Craik D. J. Cyclotides as grafting frameworks for protein engineering and drug design applications. Biopolymers 100, 480–491 (2013). 23893608 10.1002/bip.22284

[b16] Northfield S. E. . Disulfide-rich macrocyclic peptides as templates in drug design. Eur. J. Med. Chem. 77, 248–257 (2014). 24650712 10.1016/j.ejmech.2014.03.011

[b17] Wong C. T. . Orally active peptidic bradykinin B1 receptor antagonists engineered from a cyclotide scaffold for inflammatory pain treatment. Angew. Chem. Int. Ed. Engl. 51, 5620–5624 (2012). 22532483 10.1002/anie.201200984

[b18] Chan L. Y. . Engineering pro-angiogenic peptides using stable, disulfide-rich cyclic scaffolds. Blood 118, 6709–6717 (2011). 22039263 10.1182/blood-2011-06-359141

[b19] Ji Y. . *In vivo* activation of the p53 tumor suppressor pathway by an engineered cyclotide. J. Am. Chem. Soc. 135, 11623–11633 (2013). 23848581 10.1021/ja405108p PMC3767463

[b20] Gunasekera S. . Engineering stabilized vascular endothelial growth factor-A antagonists: synthesis, structural characterization, and bioactivity of grafted analogues of cyclotides. J. Med. Chem. 51, 7697–7704 (2008). 19053834 10.1021/jm800704e

[b21] Welti J., Loges S., Dimmeler S. & Carmeliet P. Recent molecular discoveries in angiogenesis and antiangiogenic therapies in cancer. J. Clin. Invest. 123, 3190–3200 (2013). 23908119 10.1172/JCI70212 PMC3726176

[b22] Suich D. J. . Template-constrained cyclic peptide analogues of somatostatin: subtype-selective binding to somatostatin receptors and antiangiogenic activity. Bioorg. Med. Chem. 8, 2229–2241 (2000). 11026536 10.1016/s0968-0896(00)00135-8

[b23] Wright R. M. . Binding epitope of somatostatin defined by phage-displayed peptide libraries. Biotechnology 13, 165–169 (1995). 9634758 10.1038/nbt0295-165

[b24] Abe R. . Topical application of anti-angiogenic peptides based on pigment epithelium-derived factor can improve psoriasis. J. Dermatol. Sci. 57, 183–191 (2010). 20060688 10.1016/j.jdermsci.2009.12.010

[b25] Dawson D. W. . Pigment epithelium-derived factor: a potent inhibitor of angiogenesis. Science 285, 245–248 (1999). 10398599 10.1126/science.285.5425.245

[b26] Maik-Rachline G. & Seger R. Variable phosphorylation states of pigment-epithelium-derived factor differentially regulate its function. Blood 107, 2745–2752 (2006). 16322471 10.1182/blood-2005-06-2547

[b27] Bae D. G., Gho Y. S., Yoon W. H. & Chae C. B. Arginine-rich anti-vascular endothelial growth factor peptides inhibit tumor growth and metastasis by blocking angiogenesis. J. Biol. Chem. 275, 13588–13596 (2000). 10788475 10.1074/jbc.275.18.13588

[b28] Yoo S. A. . Arginine-rich anti-vascular endothelial growth factor (anti-VEGF) hexapeptide inhibits collagen-induced arthritis and VEGF-stimulated productions of TNF-alpha and IL-6 by human monocytes. J. Immunol. 174, 5846–5855 (2005). 15843589 10.4049/jimmunol.174.9.5846

[b29] Podar K. & Anderson K. C. The pathophysiologic role of VEGF in hematologic malignancies: therapeutic implications. Blood 105, 1383–1395 (2005). 15471951 10.1182/blood-2004-07-2909

[b30] Weis S. M. & Cheresh D. A. alphaV integrins in angiogenesis and cancer. Cold Spring Harb. Perspect. Med. 1, a006478 (2011). 22229119 10.1101/cshperspect.a006478 PMC3234453

[b31] Adams R. L., Adams I. P., Lindow S. W., Zhong W. & Atkin S. L. Somatostatin receptors 2 and 5 are preferentially expressed in proliferating endothelium. Br. J. Cancer 92, 1493–1498 (2005). 15812556 10.1038/sj.bjc.6602503 PMC2362009

[b32] Atkins M., Jones C. A. & Kirkpatrick P. Sunitinib maleate. Nat. Rev. Drug Discov. 5, 279–280 (2006). 16628834 10.1038/nrd2012

[b33] Fosgerau K. & Hoffmann T. Peptide therapeutics: current status and future directions. Drug Discov. Today 20, 122–128 (2015). 25450771 10.1016/j.drudis.2014.10.003

[b34] Ovadia O. . Improvement of drug-like properties of peptides: the somatostatin paradigm. Expert Opin. Drug Discov. 5, 655–671 (2010). 22823205 10.1517/17460441.2010.493935

[b35] Li H. . Triazine-based tool box for developing peptidic PET imaging probes: syntheses, microfluidic radiolabeling, and structure-activity evaluation. Bioconjug. Chem. 25, 761–772 (2014). 24661266 10.1021/bc500034n PMC3993951

[b36] Gibbons D. L. & Byers L. A. A. HER 1-2 punch: dual EGFR targeting deals resistance a deadly blow. Cancer Discov. 4, 991–994 (2014). 25185188 10.1158/2159-8290.CD-14-0791 PMC4990388

[b37] Conibear A. C. . The cyclic cystine ladder of theta-defensins as a stable, bifunctional scaffold: a proof-of-concept study using the integrin-binding RGD motif. ChemBioChem 15, 451–459 (2014). 24382674 10.1002/cbic.201300568

[b38] Huang Y. H. . Design of substrate-based BCR-ABL kinase inhibitors using the cyclotide scaffold. Sci. Rep. 5, 12974 (2015). 26264857 10.1038/srep12974 PMC4532999

[b39] Sun L. C. & Coy D. H. Somatostatin receptor-targeted anti-cancer therapy. Curr. Drug Deliv. 8, 2–10 (2011). 21034425 10.2174/156720111793663633

[b40] Craword S. E., Fitchev P., Veliceasa D. & Volpert O. V. The many facets of PEDF in drug discovery and disease: a diamond in the rough or split personality disorder? Expert Opin. Drug Discov. 8, 769–792 (2013). 23642051 10.1517/17460441.2013.794781

[b41] Rai U., Thrimawithana T. R., Valery C. & Young S. A. Therapeutic uses of somatostatin and its analogues: current view and potential applications. Pharmacol. Ther. 152, 98–110 (2015). 25956467 10.1016/j.pharmthera.2015.05.007

[b42] Becerra S. P. & Notario V. The effects of PEDF on cancer biology: mechanisms of action and therapeutic potential. Nat. Rev. Cancer 13, 258–271 (2013). 23486238 10.1038/nrc3484 PMC3707632

[b43] Zhang K., Zhang L. & Weinreb R. N. Ophthalmic drug discovery: novel targets and mechanisms for retinal diseases and glaucoma. Nat. Rev. Drug Discov. 11, 541–559 (2012). 22699774 10.1038/nrd3745

[b44] Chan L. Y. . Isolation and characterization of peptides from Momordica cochinchinensis seeds. J. Nat. Prod. 72, 1453–1458 (2009). 19711988 10.1021/np900174n

[b45] Chan L. Y., Craik D. J. & Daly N. L. Cyclic thrombospondin-1 mimetics: grafting of a thrombospondin sequence into circular disulfide-rich frameworks to inhibit endothelial cell migration. Biosci. Rep. 35, e00270 (2015). 26464514 10.1042/BSR20150210 PMC4660582

[b46] Vranken W. F. . The CCPN data model for NMR spectroscopy: development of a software pipeline. Proteins 59, 687–696 (2005). 15815974 10.1002/prot.20449

[b47] Wüthrich K. NMR of Proteins and Nucleic Acids (Wiley-Interscience, 1986).

[b48] Wishart D. S. . 1H, 13C and 15N chemical shift referencing in biomolecular NMR. J. Biomol. NMR 6, 135–140 (1995). 8589602 10.1007/BF00211777

[b49] Koradi R., Billeter M. & Wuthrich K. MOLMOL: a program for display and analysis of macromolecular structures. J. Mol. Graph. 14, 51–55, 29–32 (1996). 8744573 10.1016/0263-7855(96)00009-4

