# Supplementary material for: Dual-targeting anti-angiogenic cyclic peptides as potential drug leads for cancer therapy
Source: Sci Rep. 2016 Oct 13;6:35347. doi: 10.1038/srep35347 (PMC5062114; doi:10.1038/srep35347)
Supplement: Supplementary Information [file srep35347-s1.pdf]

Supplementary Data

Dual-targeting anti-angiogenic cyclic peptides as potential drug leads for cancer therapy

Lai Yue Chan,[1] David J. Craik,[1*] and Norelle L. Daly[2*]

[1]The University of Queensland, Institute for Molecular Bioscience, 4072 Brisbane, Australia; [2]Centre for Biodiscovery and Molecular Development of Therapeutics, Australian Institute of Tropical Health and Medicine, James Cook University, 4870 Cairns, Australia

[*]To whom correspondence should be addressed:

Prof. David J. Craik
Address: The University of Queensland, Institute for Molecular Bioscience, 4072 Brisbane, Australia
Email: d.craik@imb.uq.edu.au
Phone: +61-7-3346 2019
Fax: +61-7-33462101

'or'

Prof. Norelle L. Daly
Address: Centre for Biodiscovery and Molecular Development of Therapeutics, Australian Institute of Tropical Health and Medicine, James Cook University, 4870 Cairns, Australia
Email: norelle.daly@jcu.edu.au
Phone: +61-7-4232 1815

Table S1. Molecular masses of all peptides in this study.

Peptides	Calculated Mass (Da)	Observed Mass (Da)
polyR	927.1	927.6
SST-01	594.7	595.2
SST-02	923.1	923.4
PEDF	918.0	918.2
SFTI-1	1513.8	1513.7
SFTI-polyR-L1	1799.2	1800.1
SFTI-polyR-L2	1850.3	1849.8
SFTI-SST-01	1517.9	1517.7
SFTI-SST-02	1846.2	1845.9
SFTI-PEDF	1841.2	1841.1
MCoTI-II	3452.9	3452.4
MCo-polyR-L1	3654.1	3653.8
MCo-polyR-L6	3859.6	3859.6
MCo-SST-01	3638.3	3638.2
MCo-SST-02	3741.4	3741.8
MCo-PEDF	3736.4	3736.4
MCoAA-01	3953.5	3953.0
MCoAA-02	4035.8	4035.4

Table S2. NMR Chemical shifts (ppm) of MCoAA-02 at 298K, pH 5.5

Position	Residue	HN	Hα	Hβ	Others
1	Cys	7.82	5.16	2.8	
2	Pro		4.34	1.78, 2.22	$_{\gamma}CH_2$ 1.84, 1.92; $_{\delta}CH_2$ 3.75, 3.75
3	Lys	8.15	4.19	1.62, 1.77	$_{\gamma}CH_2$ 1.31; $_{\delta}CH_2$ 1.39; $_{\varepsilon}CH_2$ 2.87
4	Ile	7.58	4.18	1.69	$_{\gamma}CH_2$ 1.27; $_{\gamma}CH_3$ 0.98; $_{\delta}CH_3$ 0.73
5	Leu	8.48	4.30	1.54, 1.54	$_{\gamma}H$ 1.43; $_{\delta}CH_3$ 0.73, 0.8
6	Lys	8.24	4.32	1.51, 1.64	$_{\gamma}CH_2$ 1.32; $_{\delta}CH_2$ 1.46; $_{\varepsilon}CH_2$ 2.72
7	Lys	8.26	4.04	1.63, 1.71	$_{\gamma}CH_2$ 1.31; $_{\delta}CH_2$ 1.52; $_{\varepsilon}CH_2$ 2.98
8	Cys	7.90	4.78	2.85, 3.11	
9	Arg	9.22	4.33	1.66, 1.75	$_{\gamma}CH_2$ 1.53; $_{\delta}CH_2$ 3.09
10	Arg	7.91	4.51	1.66, 1.84	$_{\gamma}CH_2$ 1.3, 1.41; $_{\delta}CH_2$ 2.98
11	Asp	8.94	3.99	2.59, 2.74	
12	Ser	8.03	4.20	3.73, 3.83	
13	Asp	8.45	4.50	2.70, 2.70	
14	Cys	8.03	4.90	2.59, 2.72	
15	Pro		4.50	1.84, 2.19	$_{\gamma}CH_2$ 1.93, 2.00; $_{\delta}CH_2$ 3.35, 3.64
16	Gly	8.37	3.75, 3.75		
17	Ala	8.20	4.51	1.36	
18	Cys	8.20	4.65	3.04, 3.18	
19	Ile	8.77	4.26		$_{\gamma}CH_2$ 0.62; $_{\gamma}CH_3$ 0.86; $_{\delta}CH_3$ 0.52
20	Cys	9.24	4.75	2.33, 2.74	
21	Arg	8.42	4.74	1.67, 1.84	$_{\gamma}CH_2$ 1.46; $_{\delta}CH_2$ 2.93
22	Tyr	7.59	4.53	2.89, 2.89	
23	Trp	8.41	4.65	3.68, 3.83	2H 7.05; 4H 7.41; 5H 7.24; 6H 7.15; 7H 7.29; NH 10.10
24	Lys	8.58	4.19	1.67, 1.84	$_{\gamma}CH_2$ 1.28; $_{\delta}CH_2$ 1.35; $_{\varepsilon}CH_2$ 3.11
25	Val	7.67	4.10	2.03	$_{\gamma}CH_3$ 0.79
26	Cys	8.65	5.18	2.65, 2.92	
27	Gly	9.60	3.73, 4.25		
28	Tyr	7.72	4.78	2.91, 3.03	
29	His	9.08	4.38	3.1, 3.21	
30	Leu	8.43	3.93	1.55, 1.55	$_{\gamma}H$ 1.36; $_{\delta}CH_3$ 0.54, 0.8
31	Asn	8.61	4.44	2.87, 2.87	
32	Gln	7.64	4.59	1.74, 1.85	$_{\gamma}CH_2$ 2.9
33	Pro		3.66	1.46, 1.93	$_{\gamma}CH_2$ 1.61, 1.85; $_{\delta}CH_2$ 3.35, 3.35
34	Phe	7.72	4.43	2.88, 2.99	
35	Gly	8.36	3.69, 3.89		

Figure legends

Figure S1. 1D spectra of all grafted peptides in SFTI-1 framework. All spectra were acquired at 298K with or without DSS (4,4-dimethyl-4-silapentane-1-sulfonic acid) as a calibration standard.

Figure S2. 1D spectra of all grafted peptides in MCoTI-II framework. All spectra were acquired at 298K with or without DSS (4,4-dimethyl-4-silapentane-1-sulfonic acid) as a calibration standard.

Figure S3. 2D TOCSY and NOESY spectra of the best active dual-targeting cyclic peptide, MCoAA-02. (**A**) NOESY spectra. (**B**) TOCSY spectra. These data were acquired at 298K on a 600 Mhz spectrometer.

Figure S4. Cell proliferation assay. (**A**) Cell proliferation studies on all linear epitopes, grafted peptides and native cyclic frameworks. (**B**) Cell proliferation studies on all drug controls used in this study. The assay was monitored after 48 hours incubation period. Data are expressed as the mean ± SD of triplicate measurements.

Figure S5. Peptide characterization data of MCoAA-02. (**A**) MCoAA-02 peptide purity check using Nexera UHPLC (Shimadzu) on 0.3 mL/min flowrate with 2% gradient of 0-50% solvent B. MCoAA-02 was observed with >95 % purity. (**B**) Mass spectrometry data of MCoAA-02.

Figure S6. Peptide cyclization of MCoAA-01 and MCoAA-02. These peptides were successfully cyclized using 0.1 M ammonium bicarbonate at pH 8.5 with 10% DMSO in 24 hours. These samples were ran on a X500R QTOF (SCIEX) system with a flowrate of 0.4 mL/min using a 6% gradient of 0-60% solvent B. (**A**) LC-MS trace of MCoAA-01. (**B**) LC-MS trace of MCoAA-02.

Figure S7. MCoAA-02 migration assay cells staining results. This diagram shows the effect of MCoAA-02 ranging from 0.01 pM to 10 µM in HUVEC migration assay. At the final stage of this assay, cells were fixed with 4% formaldehyde and destained with 10% acetic acid prior to absorbance reading on a spectrometer at 590 nm. Pictures labeled with 'FC' and 'DS' indicate fixed cells on polycarbonate inserts and destained cells on a 96-well plate, respectively. VEGF (0.3 nM) acts as the positive control.

Figure S8. Sunitinib effect on HUVECs migration and CAM assays. (**A**) Migration assay. (**B**) CAM assay. (**C**) Diagram showing sunitinib effect on blood vessels growth. Error bars indicate ± SD. A dotted line cuts through the bar graph indicates approximately 50% inhibition of blood vessels count. One-way ANOVA with Dunnett's post-test using multiple comparison test was used for statistical analysis. *P*-value represented as $****p \leq 0.0001$ is considered significant.

Figure S1

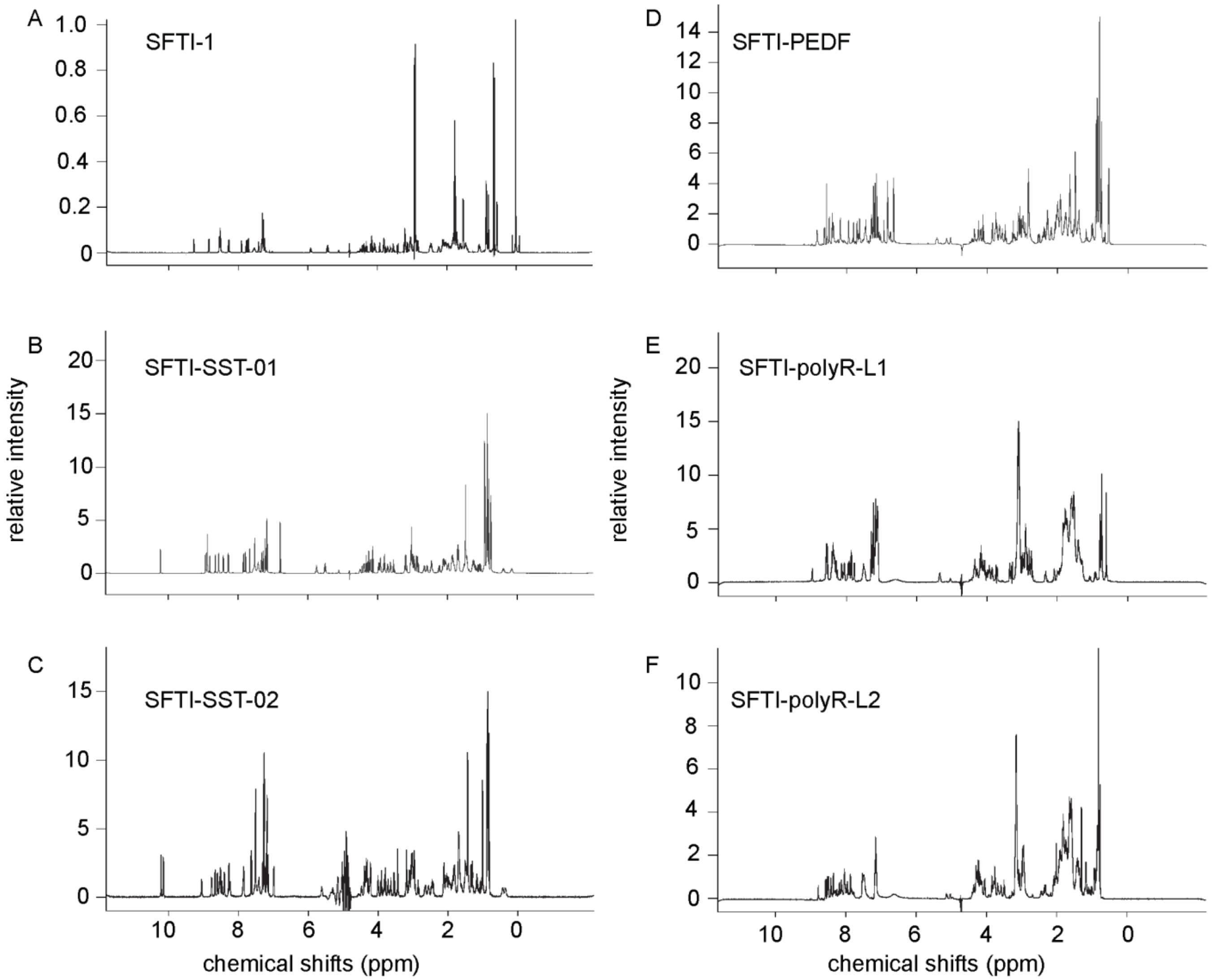
A
SFTI-1
B
SFTI-SST-01
C
SFTI-SST-02
D
SFTI-PEDF
E
SFTI-polyR-L1
F
SFTI-polyR-L2
relative intensity
chemical shifts (ppm)

Figure S2

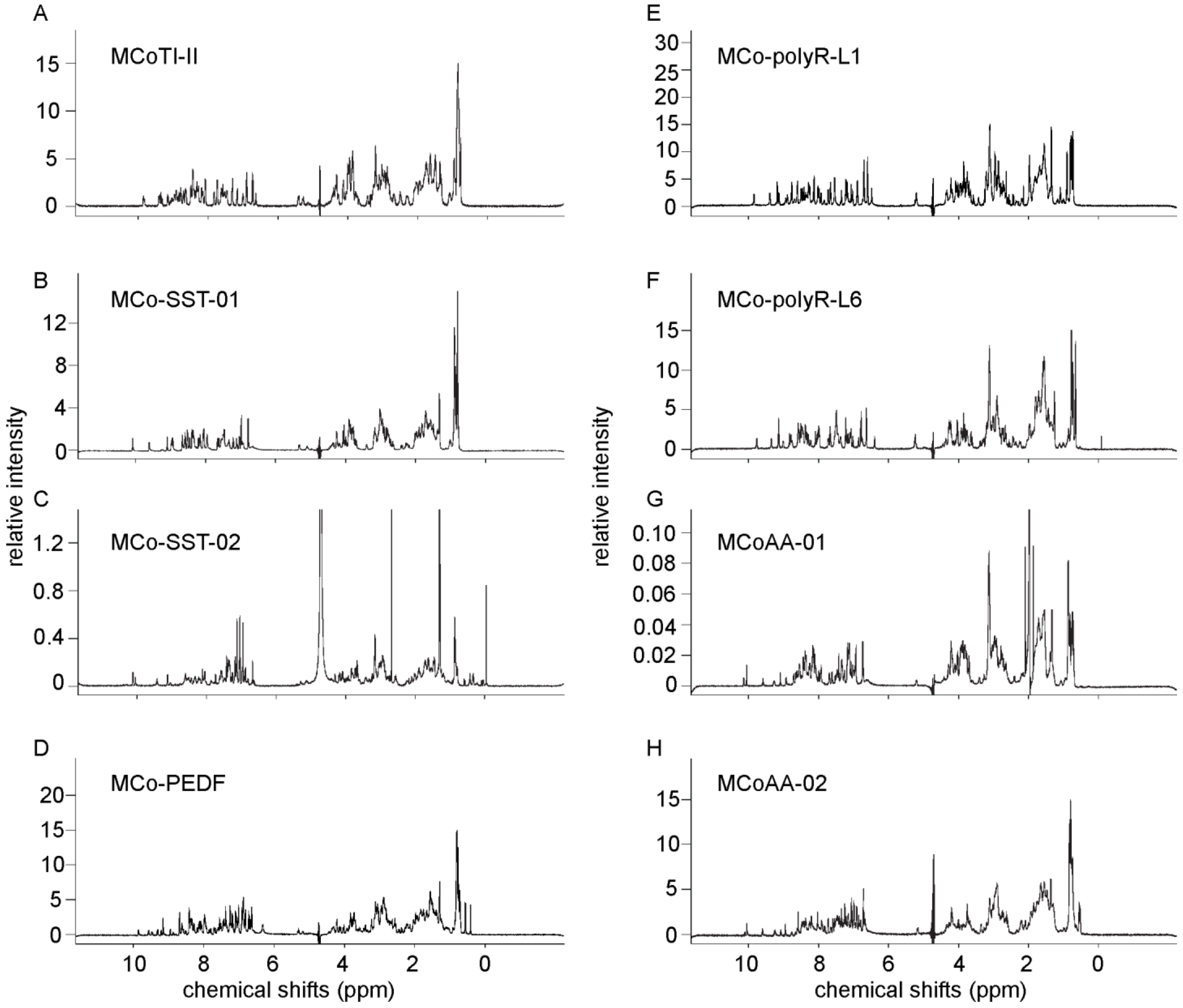

A
MCoTI-II
B
MCo-SST-01
C
MCo-SST-02
D
MCo-PEDF
E
MCo-polyR-L1
F
MCo-polyR-L6
G
MCoAA-01
H
MCoAA-02
relative intensity
chemical shifts (ppm)

Figure S3

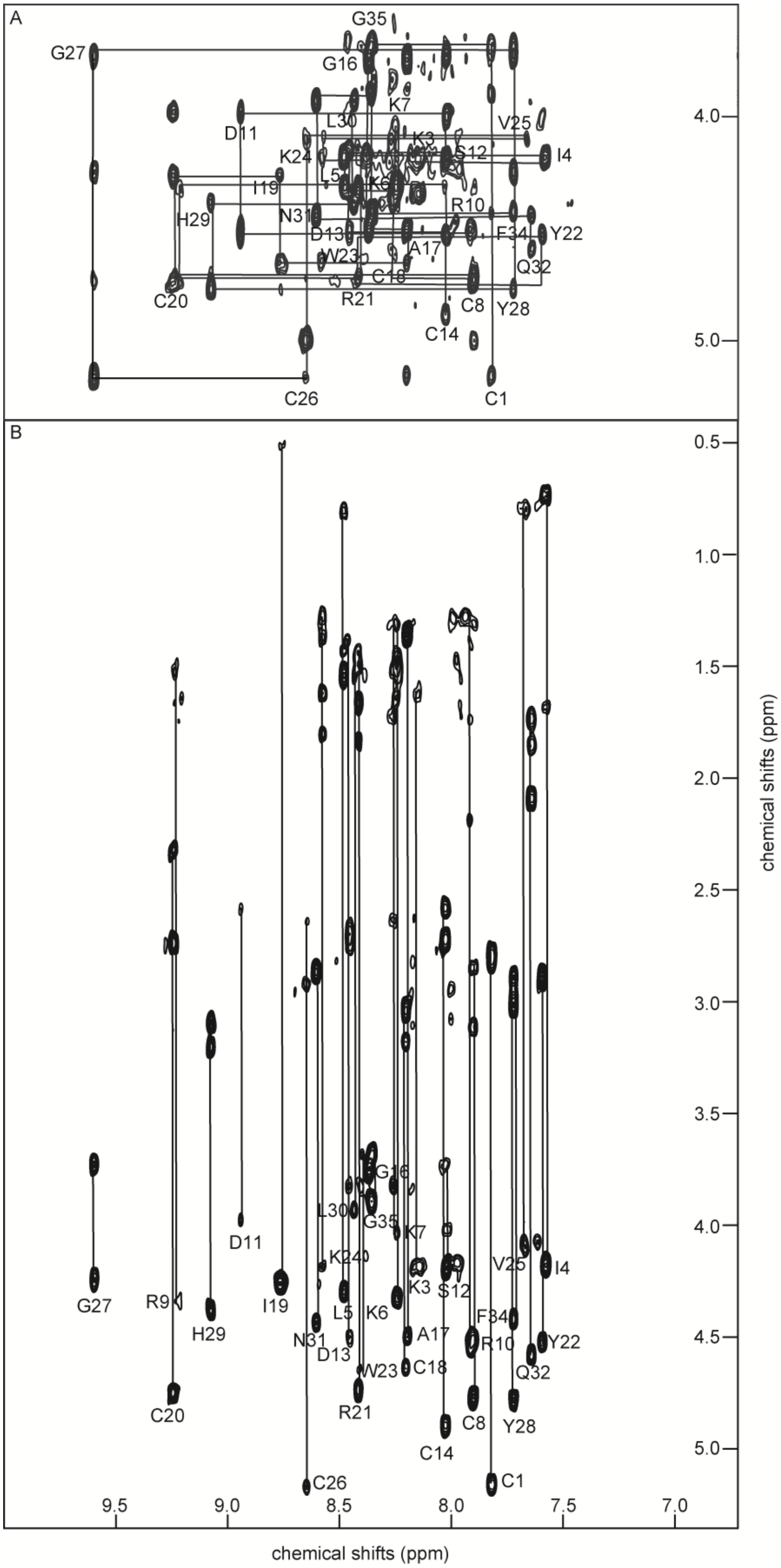
A
G35
G27
G16
K7
L30
V25
D11
K3
S12
I4
K24
L5
I19
K6
R10
H29
N31
D13
F34
Y22
A17
W23
Q32
C18
C20
R21
C8
Y28
C14
C26
C1
B
G16
L30
G35
K7
D11
K24
V25
I4
K3
S12
G27
R9
I19
H29
L5
K6
F34
N31
A17
R10
Y22
D13
W23
C18
Q32
C20
R21
C8
Y28
C14
C26
C1
4.0
5.0
0.5
1.0
1.5
2.0
2.5
3.0
3.5
4.0
4.5
5.0
9.5
9.0
8.5
8.0
7.5
7.0
chemical shifts (ppm)
chemical shifts (ppm)

Figure S4

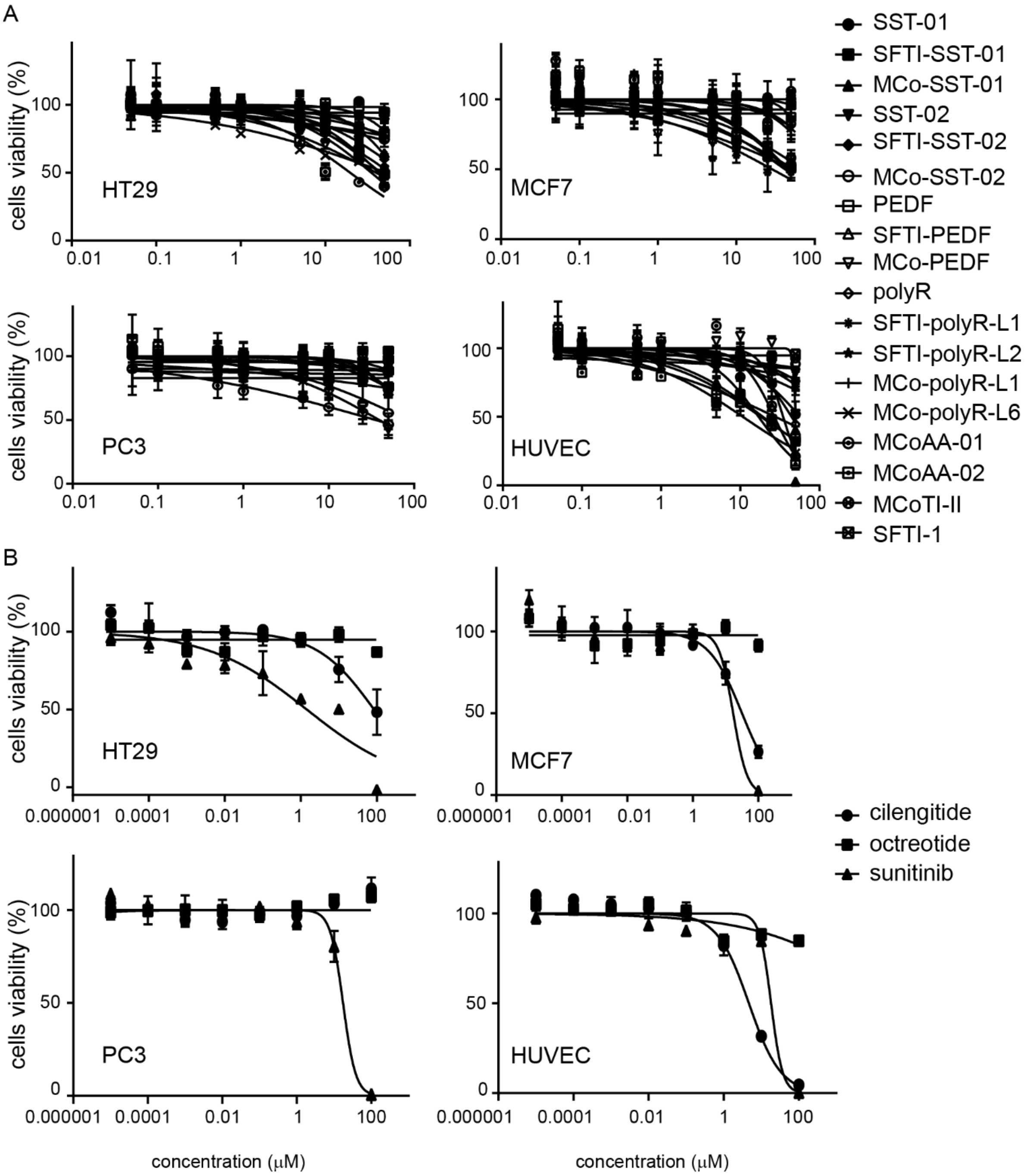
A
HT29
MCF7
PC3
HUVEC
cells viability (%)
0
50
100
0.01
0.1
1
10
100
SST-01
SFTI-SST-01
MCo-SST-01
SST-02
SFTI-SST-02
MCo-SST-02
PEDF
SFTI-PEDF
MCo-PEDF
polyR
SFTI-polyR-L1
SFTI-polyR-L2
MCo-polyR-L1
MCo-polyR-L6
MCoAA-01
MCoAA-02
MCoTI-II
SFTI-1
B
0.000001
0.0001
0.01
cilengitide
octreotide
sunitinib
concentration (μM)

Figure S5

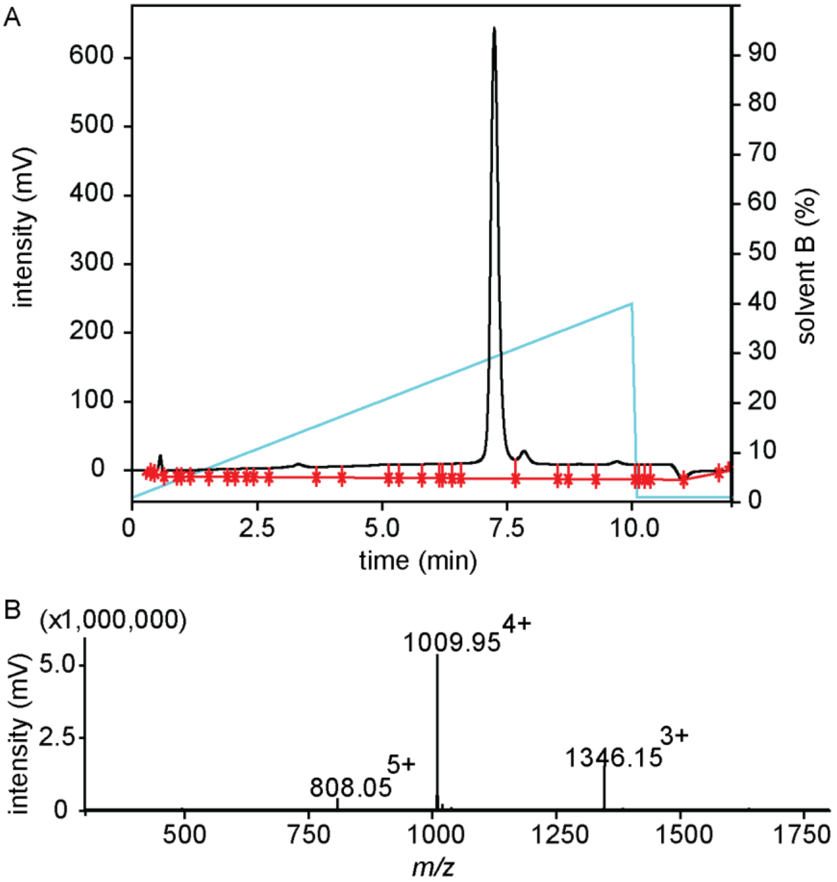
A
intensity (mV)
600
500
400
300
200
100
0
solvent B (%)
90
80
70
60
50
40
30
20
10
0
0
2.5
5.0
7.5
10.0
time (min)
B
(x1,000,000)
intensity (mV)
5.0
2.5
0
1009.95
4+
1346.15
3+
808.05
5+
500
750
1000
1250
1500
1750
m/z

Figure S6

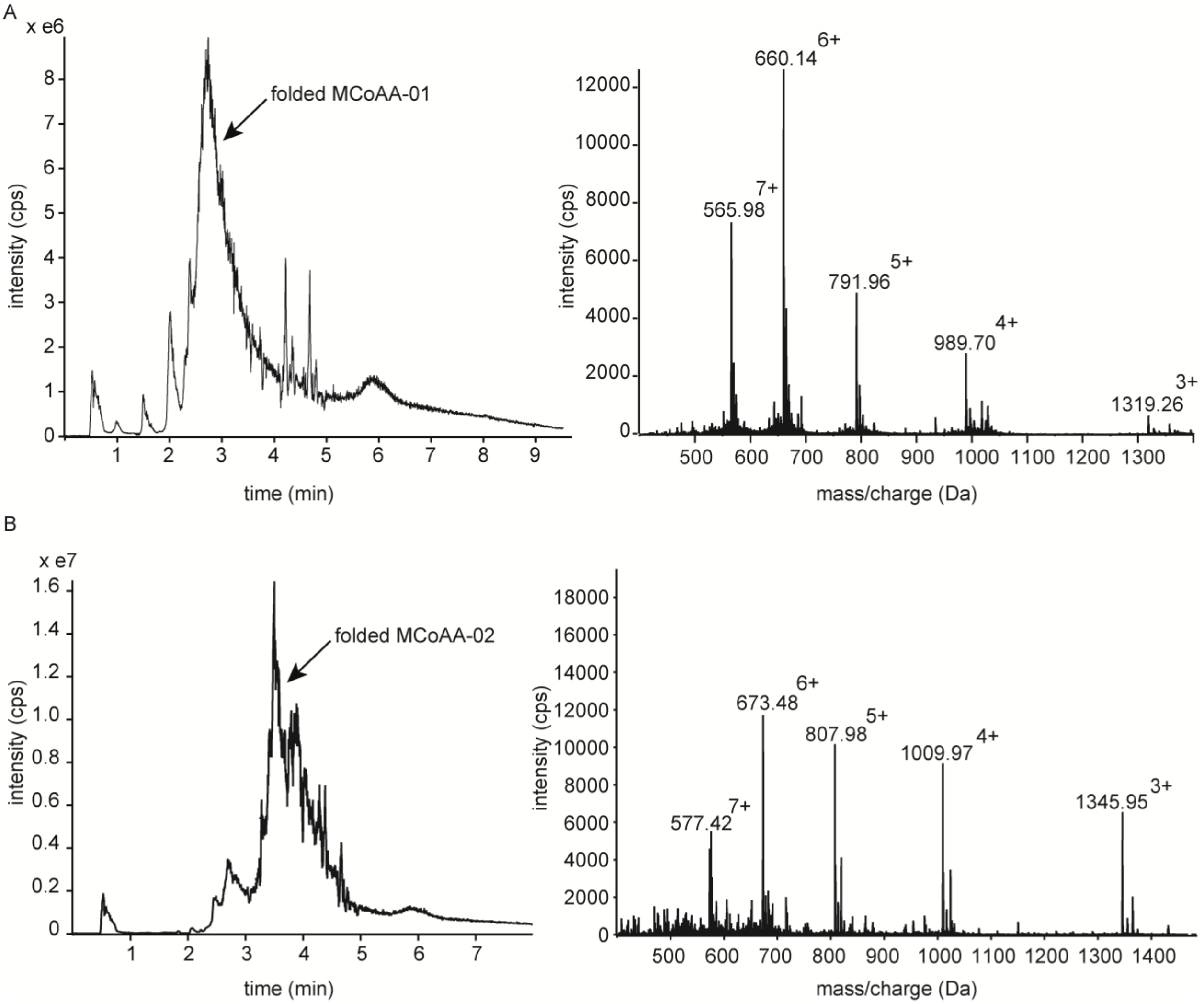
A
x e6
folded MCoAA-01
intensity (cps)
time (min)
660.14
6+
565.98
7+
791.96
5+
989.70
4+
1319.26
3+
intensity (cps)
mass/charge (Da)
B
x e7
folded MCoAA-02
intensity (cps)
time (min)
673.48
6+
807.98
5+
1009.97
4+
577.42
7+
1345.95
3+
intensity (cps)
mass/charge (Da)

Figure S7

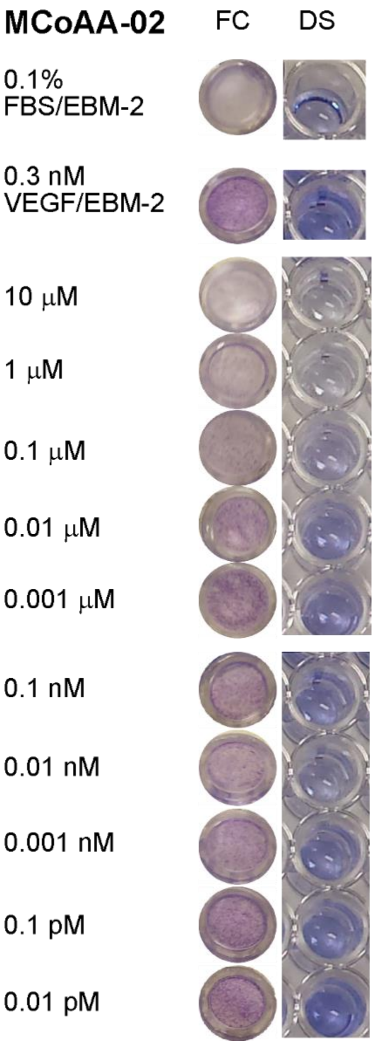
MCoAA-02
FC
DS
0.1%
FBS/EBM-2
0.3 nM
VEGF/EBM-2
10 μM
1 μM
0.1 μM
0.01 μM
0.001 μM
0.1 nM
0.01 nM
0.001 nM
0.1 pM
0.01 pM

Figure S8

A

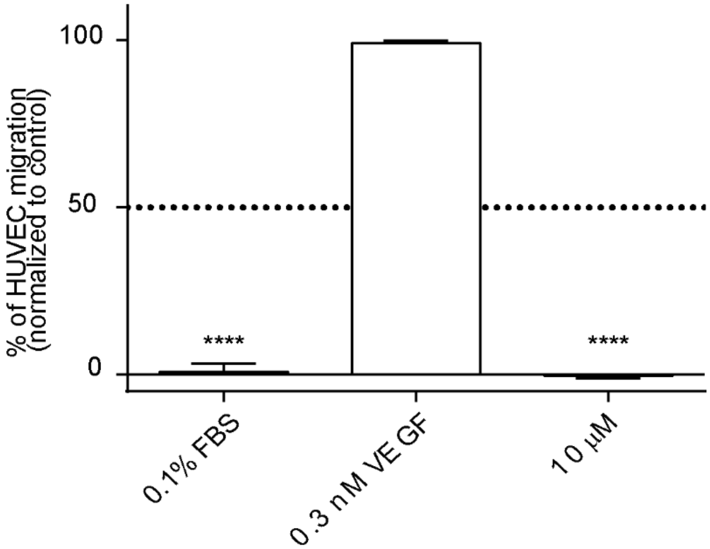

B

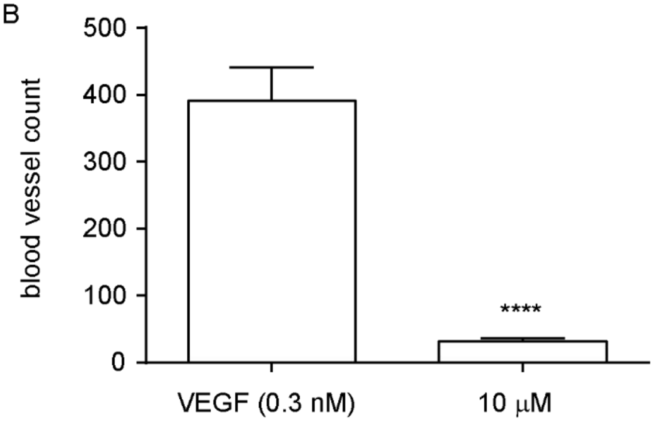

C

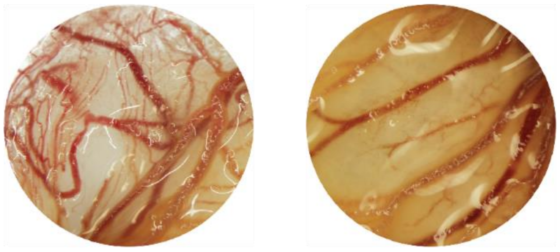

VEGF (0.3 nM) 10 μM
